# Survival in treated idiopathic normal pressure hydrocephalus

**DOI:** 10.1007/s00415-019-09598-1

**Published:** 2019-11-11

**Authors:** Kerstin Andrén, Carsten Wikkelsø, Nina Sundström, Hanna Israelsson, Simon Agerskov, Katarina Laurell, Per Hellström, Mats Tullberg

**Affiliations:** 1 Hydrocephalus Research Unit, Department of Clinical Neuroscience, Institute of Neuroscience and Physiology, The Sahlgrenska Academy, University of Gothenburg, Blå Stråket 7, 413 45 Gothenburg, Sweden; 2 Department of Radiation Sciences, Biomedical Engineering, Umeå University, Umeå, Sweden; 3 Department of Pharmacology and Clinical Neuroscience, Umeå University, Umeå, Sweden; 4 Department of Neuroscience, Uppsala University, Uppsala, Sweden

**Keywords:** Hydrocephalus, Gait disorders, Cognitive disorders, Prognosis, Cohort studies

## Abstract

**Objective:**

To describe survival and causes of death in 979 treated iNPH patients from the Swedish Hydrocephalus Quality Registry (SHQR), and to examine the influence of comorbidities, symptom severity and postoperative outcome.

**Methods:**

All 979 patients operated for iNPH 2004–2011 and registered in the SHQR were included. A matched control group of 4890 persons from the general population was selected by Statistics Sweden. Data from the Swedish Cause of Death Registry was obtained for patients and controls.

**Results:**

At a median 5.9 (IQR 4.2–8.1) year follow-up, 37% of the iNPH patients and 23% of the controls had died. Mortality was increased in iNPH patients by a hazard ratio of 1.81, 95% CI 1.61–2.04, *p* < 0.001. More pronounced symptoms in the preoperative ordinal gait scale and the Mini-mental State Examination were the most important independent predictors of mortality along with the prevalence of heart disease. Patients who improved in both the gait scale and in the modified Rankin Scale postoperatively (*n* = 144) had a similar survival as the general population (*p* = 0.391). Deaths due to cerebrovascular disease or dementia were more common in iNPH patients, while more controls died because of neoplasms or disorders of the circulatory system.

**Conclusions:**

Mortality in operated iNPH patients is 1.8 times increased compared to the general population, a lower figure than previously reported. The survival of iNPH patients who improve in gait and functional independence is similar to that of the general population, indicating that shunt surgery for iNPH, besides improving symptoms and signs, can normalize survival.

## Introduction

Idiopathic Normal Pressure Hydrocephalus (iNPH) is a treatable and under-diagnosed disorder that affects 2–4% of persons aged 65 years or older [1–4]. INPH patients present with gait and balance difficulties, cognitive dysfunction, and urinary incontinence [5]. The treatment is surgical, by insertion of a CSF diverting shunt system, which improves more than 80% of the patients on a short-term basis [6].

Survival in untreated iNPH patients is substantially reduced, with a hazard ratio (HR) for death of 3.8 compared to the general population [7]. In treated iNPH patients, a relative risk (RR) for death of 3.3 [8] and a standardized mortality ratio (SMR) of 2.5 [9] have been calculated in single center studies—similar to the mortality of first-time stroke sufferers [8]. Cardiovascular and cerebrovascular diseases have been reported as common causes of death in iNPH patients [8–10], but this has not been thoroughly explored or compared to the general population.

Risk factors for cerebrovascular disease, as well as comorbidity of cardio- and cerebrovascular diseases are common in iNPH [11–13], but it is unknown to what extent these factors influence survival in these patients. Furthermore, the association between the severity of symptoms and survival in iNPH patients has not been studied, and whether the degree of improvement post-shunting influences survival is largely unknown.

The aim of this study was to describe survival and causes of death in a large cohort of unselected iNPH patients from the Swedish Hydrocephalus Quality Registry (SHQR), and how vascular comorbidities, preoperative symptom severity, and response to surgery influence survival.

## Methods

Data on a cohort of 979 iNPH patients operated 2004–2011 in 5 of the 6 neurosurgical centers in Sweden was extracted from the SHQR on the 1st of September 2014, see Table 1 for baseline characteristics. The cohort has been described previously [14].

**Table 1:** Baseline characteristics for the 979 patients operated for iNPH 2004–2011

	iNPH patients, *n* = 979
Demography	
Age (years), median (IQR)	74 (68–78)
Sex, female, *n* (%)	413 (42)
Symptom grading scales	Median (IQR)
Gait scale (*n* = 835)	4 (3–6)
Balance scale (*n* = 747)	3 (3–5)
Continence scale (*n* = 814)	3 (2–4)
MMSE (*n* = 737)	25 (20–28)
mRS (*n* = 755)	2 (2–3)
Vascular comorbidity	*n* (%)
Hypertension (*n* = 891)	438 (49)
Diabetes Mellitus (*n* = 887)	189 (21)
History of stroke (*n* = 874)	119 (14)
Heart disease (*n* = 892)	231 (26)
Claudication (*n* = 458)	7 (1.5)
Number of vascular comorbidities	*n* (%)
0	372 (38)
1	316 (32)
2	205 (21)
3	74 (7.6)
4	9 (0.9)
5	0
None available	3 (0.3)
Number of comorbidities, median (IQR)	1 (0–2)
Type of primary surgery, *n*	*n*
Shunt	974
VP/VA/not specified	953/6/15
Ventriculostomy	5^a^

^a^Three out of five patients first operated with ventriculostomy were re-operated with shunt insertion (after 2 weeks, 6 weeks and 8 months)

*INPH* idiopathic normal pressure hydrocephalus, *IQR* interquartile range, *MMSE* mini-mental state examination, *mRS* modified Rankin Scale

### Clinical symptom grading

Patients’ symptoms were graded preoperatively and three months postoperatively using five different scales: ordinal scales for gait, balance and continence [15] (Table 2), the modified Rankin scale (mRS, score 0–5) [16, 17], and the mini-mental state examination (MMSE, score 0–30) [18] (Table 1).

**Table 2:** Clinical symptom grading scales

Score	Gait	Balance	Continence	mRS
0				No symptoms
1	Normal	Stands independently ≥ 30 s on either lower extremity alone	Normal	No significant disability. Able to carry out all usual activities, despite some symptoms
2	Slight disturbance of tandem walk and turning	Stands independently for 5–29 s on either lower extremity alone	Urgency without incontinence	Slight disability. Able to look after own affairs without assistance, but unable to carry out all previous activities
3	Wide-based gait with sway, without foot corrections	Stands independently ≥ 30 s with the feet together (at the heels)	Infrequent incontinence without napkin	Moderate disability. Requires some help, but able to walk unassisted
4	Tendency to fall, with foot corrections	Stands independently 5–29 s with feet together (at the heels)	Frequent incontinence with napkin	Moderately severe disability. Unable to attend to own bodily needs without assistance, and unable to walk unassisted
5	Walking with cane	Stands independently ≥ 30 s with the feet apart (one foot length)	Bladder incontinence	Severe disability. Requires constant nursing care and attention, bedridden, incontinent
6	Bi-manual support needed	Stands independently 5–29 s with the feet apart (one foot length)	Bladder and bowel incontinence	
7	Aided	Unable to stand without assistance	Indwelling urinary catheter	
8	Wheelchair bound			

The 3 months postoperative results in each of the five scales were graded as “improved” for patients with improvement by at least 1 point; “unchanged” for patients with unchanged results; and “deteriorated” for patients, whose score had deteriorated at least 1 point.

### Control group

A control group from the general population was defined by the governmental bureau Statistics Sweden. Five control persons were matched to each patient with regard to sex, habitational municipality, and age at the first surgical treatment for iNPH (*n* = 4890). For one patient, it was not possible to find any matching controls.

### Mortality and causes of death

For deceased patients and controls, information on dates and causes of death until the 31st of December 2014 was commissioned from the Swedish Cause of Death Registry (CDR), governed by the National Board of Health and Welfare. In Sweden, diagnoses (single or multiple) on death certificates are written by physicians in text, which is then transferred to ICD-10 diagnostic codes by the National Board of Health and Welfare. An internationally used algorithm [19] is then applied to determine the underlying cause of death; this diagnosis is reported here.

The underlying causes of death were categorized into groups mainly based on ICD-10 chapters A-Y, as shown in Fig. 3.

### Statistics

The Mann–Whitney *U* test was used for comparisons between two groups. Kruskal–Wallis test was used for comparisons between > 2 groups. Fisher’s exact test was used for comparing proportions between two groups and Pearson Chi square for comparing proportions between > 2 groups. The median follow-up time was calculated with the use of the reverse Kaplan–Meier method [20].

Survival analyses were performed by means of the Kaplan–Meier method, and between-group comparisons were investigated with the log-rank test. Furthermore, survival analyses were performed by Cox proportional hazards models; in multivariable models, a forward stepwise approach was applied with rejection of variables not reaching below the 0.05 significance level. The proportional hazards assumption was assessed by goodness-of-fit tests and visual analysis of plots of scaled Schoenfeld residuals against time.

The ordinal symptom grading scales for preoperative gait, balance, continence, and mRS were dichotomized before entry into Cox models. The cutoffs for dichotomization of the scales were set at the medians, which were also considered clinically relevant levels (for the gait scale ≥ 5 signifying walk with or without gait aids, for the balance and the continence scales ≥ 4, and for the mRS the cutoff was ≥ 3 signifying independent living or not).

Time-to-event for survival analysis of preoperative variables was calculated from the date of surgery. Time-to-event for survival analysis based on data from the 3 month postoperative control was recalculated by resetting the starting point to the date of postoperative follow-up. In both cases, the patients’ dates of surgery and of postoperative follow-up, respectively, were used for the corresponding control persons.

Causes of death were presented as proportionate mortality ratios, and compared by Fisher’s exact test. Statistical significance was set at the 0.05-level. All analyses were performed with IBM SPSS 24.0 for Windows or Stata 14.0 IC.

## Results

During the follow-up period of a median of 5.9 years (interquartile range, IQR 4.2–8.1), 37% (*n* = 358) of the iNPH patients, and 23% (*n* = 1101) of the control group died. The estimated 5-year-survival was 69% for iNPH patients compared to 82% for controls and mortality was increased in iNPH patients by HR 1.81 (95% CI 1.61–2.04, *p* < 0.001). The event rate for iNPH patients was 74 observed events per 1000 person-years, compared to 41 for controls.

The 30-day postoperative mortality rate was 0.5% (*n* = 5) [14]. Counting 30 days from the patients’ date of surgery for the corresponding controls, 0.4% of the controls died (*n* = 22), and there was no significant difference between patients and controls (*p* = 0.80).

### Preoperative characteristics and survival

**Fig. 1:**
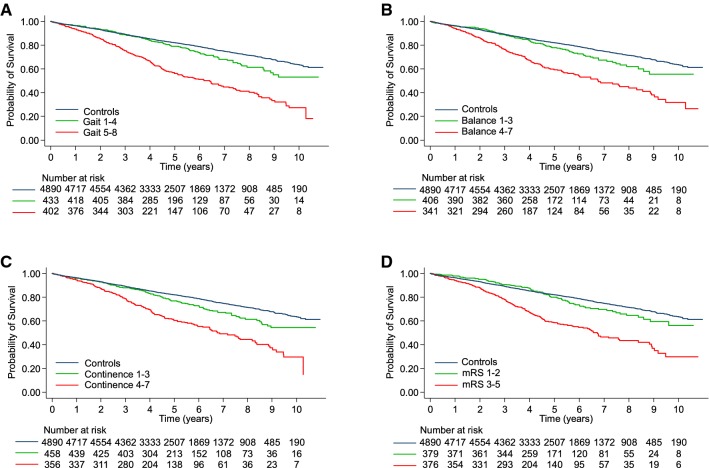
Survival in relation to preoperative symptom grading. Kaplan–Meier plots of survival in iNPH patients grouped according to dichotomized ordinal symptom grading of preoperative: **a** gait scale, **b** balance scale, **c** continence scale, and **d** the modified Rankin scale (mRS). Log-rank test: *p* < 0.001 for patients in the higher vs lower part of the scales in A-D

Higher age, male sex, prevalence of previous strokes or of any heart disease, and a higher number of comorbidities at the time of diagnosis were all significantly associated with shorter survival in iNPH patients. Furthermore, shorter survival was seen in patients with more pronounced symptomatology at baseline, in all symptom grading scales (Table 3, Fig. 1).

**Table 3:** Preoperative variables' effect on mortality

	Probability of survival at 5 years, %		Univariable Cox regression			Multivariable Cox regression, significant covariates in model		
			HR	95% CI	*p*	HR	95% CI	*p*
Age/10			1.99	1.70–2.33	< 0.001	2.01	1.63–2.46	< 0.001
	*Male*	*Female*						
Sex (male)	65	75	1.36	1.10–1.69	0.005	1.37	1.03–1.82	0.031
	*Yes*	*No*						
Hypertension	68	70	1.10	0.89–1.37	0.382	Not included		
Diabetes	66	69	1.18	0.92–1.53	0.197	Not included		
Stroke	56	71	1.54	1.17–2.04	0.002	ns		
Heart disease	57	73	1.66	1.32–2.09	< 0.001	1.59	1.19–2.12	0.002
Claudication	54	72	1.91	0.60–6.02	0.271	Not included		
Number of comorbidities			1.22	1.11–1.36	< 0.001	ns		
	*Yes*	*No*						
Gait scale ≥ 5	56	79	2.20	1.75–2.77	< 0.001	1.78	1.34–2.36	< 0.001
Balance scale ≥ 4	57	77	1.98	1.54–2.53	< 0.001	ns		
Continence scale ≥ 4	60	77	1.87	1.49–2.36	< 0.001	ns		
mRS ≥ 3	58	80	2.23	1.73–2.87	< 0.001	ns		
MMSE score/5			0.67	0.60–0.75	< 0.001	0.77	0.68–0.88	< 0.001

*MMSE* mini-mental state examination, *mRS* modified Rankin Scale

Including these significant factors into a multivariable model, characteristics shown to be independently associated with mortality were: higher age, male sex, having a heart disease (59% higher), being scored 5–8 on the gait scale (78% higher) and the MMSE (23% lower mortality per five points higher score) (Table 3).

In addition, a higher HR was found step by step for each score in the scales of gait (scores 1–8: HR 1.34–5.12), balance (scores 1–7: 3.28–8.38), continence (scores 1–6: HR 0.74–2.04), and in the mRS (scores 1–5 were represented preoperatively: HR 1.42–5.76). Only in the continence scale, the linearity was not complete throughout the scale: a score of 7 had an HR of 1.99, whereas a score of 6 had an HR of 2.04.

### Postoperative outcome and survival

Postoperative improvement in the gait scale or in the mRS was associated with better survival (HR 0.57, 95% CI 0.44–0.75 and HR 0.55, 95% CI 0.41–0.75, respectively, *p* < 0.001 for both). In addition, improvement in these two scales was independent predictors of increased survival, in a multivariable model adjusted for age, sex, and heart disease (improvement in gait: HR 0.63, 95% CI 0.45–0.89, *p* = 0.007 and improvement in mRS: 0.67, 95% CI 0.47–0.89, *p* = 0.019). No significant effects on survival were seen regarding postoperative improvement in the scales for balance or continence, nor in the MMSE (*p* = 0.154, *p* = 0.111, and *p* = 0.46, respectively).

**Fig. 2:**
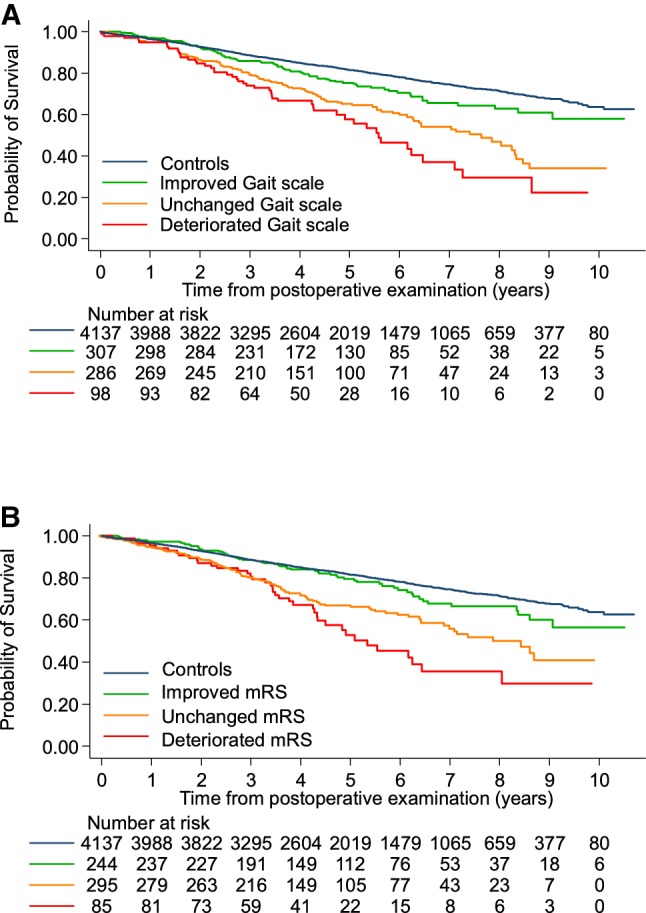
Survival in relation to postoperative development on the Gait scale and on the mRS. Kaplan–Meier plots of survival in iNPH patients after postoperative follow-up, with separate lines for patients who were improved, unchanged or deteriorated in **a** gait scale and **b** modified Rankin scale (mRS). Log-rank test: *p* < 0.001 for both

Next, survival among patients was compared to survival among the controls. The results are presented in Table 4 and Fig. 2. The survival of the 144 patients who improved in both the gait scale and in the mRS did not differ significantly from the survival of the controls (Table 4).

**Table 4:** Survival in relation to postoperative development in the gait scale and in the mRS

	Adjusted HR^a^	95% CI	*p*
Controls	Reference		
Improved gait scale (*n* = 307)	1.43	1.14–1.80	0.002
Unchanged gait scale (*n* = 286)	2.09	1.71–2.54	< 0.001
Deteriorated gait scale (*n* = 98)	2.69	2.00–3.60	< 0.001
Controls	Reference		
Improved mRS (*n* = 244)	1.30	1.00–1.69	0.047
Unchanged mRS (*n* = 295)	1.82	1.49–2.23	< 0.001
Deteriorated mRS (*n* = 85)	2.62	1.90–3.62	< 0.001
Controls	Reference		
Both gait scale and mRS improved (*n* = 144)	1.16	0.83–1.64	0.391
One of gait scale or mRS improved (*n* = 198)	1.51	1.13–2.01	0.005
Gait scale not improved, mRS not improved (*n* = 233)	2.30	1.87–2.83	< 0.001

*mRS* modified Rankin Scale

^a^HR adjusted for age and sex

### Causes of death

**Fig. 3:**
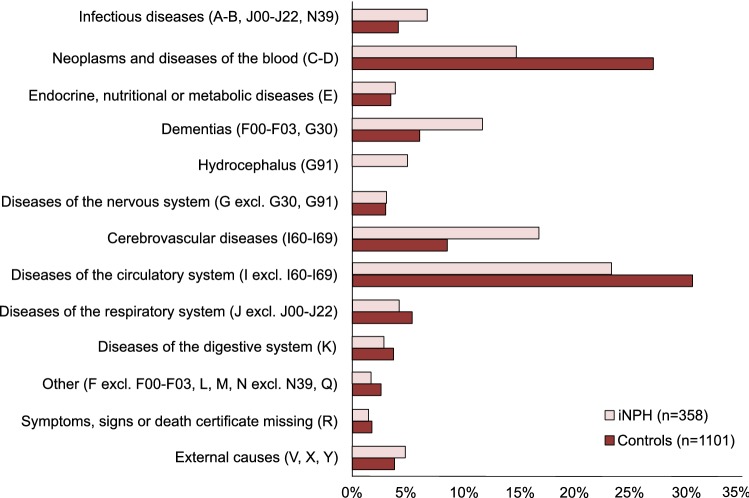
Underlying causes of death in iNPH patients and controls. Proportionate mortality ratios in 358 iNPH patients compared to 1101 controls. ICD-10 diagnostic code chapters for each category are presented within parenthesis. **p < 0.01, ***p < 0.001

Deaths due to cerebrovascular disease or dementia were more common in iNPH patients than in controls (17 vs 8.5%, *p* < 0.001 and 12 vs 6.0%, *p* = 0.001) (Fig. 3). More controls died because of neoplasms and of disorders of the circulatory system (15 vs 27%, *p* < 0.001 and 23 vs 31%, *p* = 0.009) (Fig. 3). A diagnosis of hydrocephalus was coded as the underlying cause of death in 5% of the patients but not in any controls (*p* < 0.001).

Looking at external causes of death, there was no significant difference in deaths caused by falls, with 13 (3.6%) cases in iNPH patients and 27 (2.5%) in controls, *p* = 0.26. Nor were there any significant differences between the groups concerning any other specific external causes of death.

## Discussion

This is a registry study unique in its size of 979 iNPH patients with a long follow-up of up to 10 years. The aim was to describe the patients’ survival and to analyze how survival is influenced by different factors—a topic that has not been thoroughly studied previously.

The study showed a 1.8 times increased risk of death for iNPH patients compared to controls. In the group of iNPH patients, more pronounced symptomatology before shunt surgery, was associated with higher mortality. This finding applied for all tested domains—with gait and cognition (MMSE) being the most important, as shown in a multivariable model. Unexpectedly, among the reported comorbidities, only heart disease was found to be associated with higher mortality.

The patients who improved in the gait scale or in the mRS postoperatively, survived longer. In fact, the survival of patients who improved in both these scales was no different than that of the control group. The patients who continued to deteriorate postoperatively had a substantially higher mortality than patients with unchanged scores.

The most common causes of death in both iNPH and in the control group were vascular diseases and neoplasms. Deaths attributed to cerebrovascular disease or to dementia were over-represented in iNPH patients, while neoplasms or disorders of the circulatory system were more common causes of death among controls.

Two earlier studies have reported mortality in iNPH patients compared to controls from the general population, with an SMR of 2.5 calculated by Tisell et al. in 2006 [9] and a relative risk for death of 3.3 after 3 years found by Malm et al. in 2000 [8]. As different statistical methods are used, the results are not directly comparable, but the HR of 1.8 that was calculated in our study, is a lower figure than those previously reported. One reason for the lower mortality in the present study could be that with time the awareness and knowledge of iNPH has improved and the incidence of shunt surgery for iNPH has increased [21], possibly meaning that patients with less pronounced symptoms may have been operated on to a larger extent.

Furthermore, surgical and anaesthetical techniques have improved and deaths related to shunt surgery or the early postoperative period have decreased [6]. In our data, deaths related to the shunt surgery in itself are rare, with 0.5% 30-day postoperative mortality, compared to 0.9% (1 of 109) [9] and 2.4% (1 of 42) [8] in the two earlier studies mentioned above.

### Preoperative characteristics

INPH patients with more pronounced symptoms preoperatively had shorter survival. This comes as no surprise but the finding has not been described earlier. More pronounced symptoms are signs of a more extensive disease process, in iNPH, but also in other diseases. For statistical reasons, the ordinal grading scales were dichotomized, showing significant associations with mortality. In addition, for each step of the ordinal scales of gait, balance, continence, and the mRS, HR for death was higher. The fact that only the score of 7 in the continence scale stands out from this finding, perhaps further supports that the more pronounced iNPH symptoms, the higher the mortality—since the score is assigned to patients with indwelling urinary catheters; a urinary problem not typical of iNPH. The scale scores of 1–6 follow the pattern of increasing HR for each step of the scale, as in the other scales.

Of the reported comorbidities in the SHQR, only heart disease was significant in the multivariable model. Prevalence of stroke was significant only in the univariable analysis, although cerebrovascular disease was the second most common cause of death in iNPH patients. However, of patients who later died due to cerebrovascular disease, 36% had the previous strokes reported at the time of diagnosis, so in the remaining cases, strokes manifested later. A Finnish study with 283 iNPH patients found atrial fibrillation and type-2-diabetes to be independently associated with higher mortality [10]. The first finding is in line with the present study, even if the SHQR report on cardiac disease is less detailed, but the association between diabetes and increased mortality in iNPH patients, could not be confirmed (Table 3).

Even if a heavier burden of symptoms and comorbid heart disease both are associated with reduced survival, treatment for iNPH is still highly beneficial with an estimated gain of 2.2 life years and 1.7 quality-adjusted life years [22].

### Postoperative outcome

Patients who improved in the gait scale and in the mRS were shown to subsequently survive longer than patients who did not improve in these scales. A similar finding was reported by Mirzayan et al.; patients who survived the follow-up after 5 years, showed greater postoperative improvements in cardinal symptoms, than patients who died before the follow-up [23]. An earlier study showed that patients who had to wait for shunt surgery had worse surgical outcome, meaning that the reversibility of symptoms diminish over time [24]. The finding in the present study, that patients with good surgical effects had better survival or even the same as the general population, should further emphasize the need of early diagnosis and operation without delay.

Why some patients deteriorated postoperatively is not known. One hypothesis could be that a higher burden of vascular comorbidity would explain both the worse result and the higher mortality. However, analyzing the groups of patients that were improved, unchanged, or deteriorated in the gait scale and in the mRS—no difference in the prevalence of the reported comorbidities was found. This is in line with earlier studies: also patients with vascular comorbidity improve after shunting for iNPH [25, 26]. In addition, there were no sex differences and no large age differences—the median ages for patients who were improved, unchanged or deteriorated in the mRS were 73, 75 and 74 years (*p* = 0.008), and in the gait scale, there was no significant difference.

In summary, there are probably other factors not accounted for in these registry data that explain why some patients deteriorate after surgery, such as concomitant neurodegenerative disorders.

### Causes of death

Several earlier studies have shown that the most common causes of death in iNPH patients, are cerebrovascular and other cardiovascular diseases [8, 9, 26, 27]. In addition, in our study, cardiovascular and cerebrovascular diseases were the most common causes of death in iNPH patients, with a clear overrepresentation of cerebrovascular disease for iNPH patients when compared to controls. In iNPH, the prevalence of risk factors for cerebrovascular disease is higher than in controls [11, 12]. These findings lend further support to similar pathophysiological mechanisms underlying the development of iNPH and cerebrovascular disease [28]. Whether in addition, there is a synergistic effect of the hydrocephalic state in itself exacerbating the cerebrovascular disease processes remains to be elucidated.

Malignancies were the third most common cause of death in both groups, with a large difference between the groups, as it was almost twice more common in controls than in iNPH patients. There might be a negative selection bias of patients with known malignancies—care providers are not inclined to refer these patients to hydrocephalus teams and they are less likely to be offered shunt surgery. Another explanation could be that the iNPH patients live shorter lives, having less time to develop malignancies. The same two arguments could at least partly explain why death caused by cardiovascular disease was more common in the control group. Dementia was the fourth most common underlying cause of death in both iNPH patients and controls, and it was, not unexpectedly, over-represented in iNPH patients.

### Strengths and limitations

This study has three major strengths. First, the registry-based design, allowing for a uniquely large cohort of 979 patients, with a long follow-up period of up to 10 years. Prospective and extensive data collection was performed in five different hospitals, covering approximately 80% of the Swedish population [21], including all patients operated for iNPH, representing every day clinical practice.

Second, the primary outcome is survival, meaning an absolute outcome measure with no missing data, since the CDR has a complete coverage of dates of death. The use of a large matched control group, finally, constitutes the third major strength.

The limitations are also connected to the fact that this is a registry study. The quality of registrations in the different hospitals has been subject to controls regularly, but formal measures of inter-rater reliability have not been made and would hardly be possible, due to the large number of persons involved in scoring. Evidently, no examiner was blinded. For each variable, there are missing entries, as declared in Table 1f or the baseline data, with missing data ranging between 15% for the gait scale and 25% for the MMSE. For the comorbidities, the proportion of missing data is 11% or less, except for claudication, which was later included in the SHQR. For analyses regarding postoperative outcome, only patients with available pre- and postoperative scorings in each scale are included, and the numbers are reported in Table 4.

Furthermore, there are inborn limitations in the use of causes of death from the CDR. The diagnoses can be miscoded or inexact, due to difficulties assessing the actual cause of death. Especially, in older persons, often several different factors contribute to death and it can be difficult to assess which one is actually the underlying cause. The Swedish National Board of Health and Welfare applies an internationally used algorithm for this evaluation and the same method is used in all cases. Consequently, the best available data have been used, and the same problems apply for both groups, meaning that comparisons should be valid. There were only 0.8% missing death certificates in total in this study, and they are included in the R chapter (ICD-10 code R99.9).

The fact that improved or even normalized survival is seen for patients with good response to shunt surgery has important implications. It strengthens the understanding that shunt surgery is effective in iNPH, and it emphasizes the need to give persons with iNPH symptoms the chance of proper diagnosis and treatment. Physicians need to be aware of iNPH symptoms and initiate investigations to avoid missing this opportunity.

## Conclusion

In this registry study of 979 iNPH patients who underwent surgery in Sweden, mortality is increased 1.8 times compared to the general population. Preoperative symptom severity is linked to mortality, especially for gait and cognition (MMSE). Postoperative improvement in gait or in functional independence (mRS) is associated with longer survival. The survival of iNPH patients who improve in both the gait scale and in the modified Rankin Scale is similar to that of controls from the general population, indicating that shunt surgery for iNPH besides improving the symptoms and signs can normalize survival.

## Study funding

Supported by the Edit Jacobsson Foundation, the Gothenburg Medical Society, the Swedish State Support for Clinical Research (LUA-ALF) and the Gothenburg Foundation for Neurological Research (ISNF).
